# An Ethical Façade? Medical Students' Miscomprehensions of Substituted Judgment

**DOI:** 10.1371/journal.pone.0004374

**Published:** 2009-02-04

**Authors:** Farr A. Curlin, Ryan E. Lawrence, Julie Fredrickson

**Affiliations:** 1 Section of General Internal Medicine, Department of Medicine, University of Chicago, Chicago, Illinois, United States of America; 2 MacLean Center for Clinical Medical Ethics, University of Chicago, Chicago, Illinois, United States of America; 3 Pritzker School of Medicine, University of Chicago, Chicago, Illinois, United States of America; 4 The Undergraduate College, University of Chicago, Chicago, Illinois, United States of America; University of Bern, Switzerland

## Abstract

**Background:**

We studied how well first-year medical students understand and apply the concept of substituted judgment, following a course on clinical ethics.

**Method:**

Students submitted essays on one of three ethically controversial scenarios presented in class. One scenario involved a patient who had lost decisional capacity. Through an iterative process of textual analysis, the essays were studied and coded for patterns in the ways students misunderstood or misapplied the principle of substituted judgment.

**Results:**

Students correctly articulated course principles regarding patient autonomy, substituted judgment, and non-imposition of physician values. However, students showed misunderstanding by giving doctors the responsibility of balancing the interests of the patient against the interests of the family, by stating doctors and surrogates should be guided primarily by a best-interest standard, and by suggesting that patient autonomy becomes the guiding principle only when patients can no longer express their wishes.

**Conclusion:**

Students did not appear to internalize or correctly apply the substituted judgment standard, even though they could describe it accurately. This suggests the substituted judgment standard may run counter to students' moral intuitions, making it harder to apply in clinical practice.

## Introduction

Substituted judgment has become the normative criterion for making medical decisions when adult patients cannot express their wishes.[1]–[3] According to this standard, when a patient loses decisional capacity and has not provided doctors with sufficient guidance about what type of care he or she wishes to receive or forego, health care providers should identify an appropriate surrogate decision maker. They should then instruct the surrogate to make medical decisions based on their judgment of what the patient would most likely have chosen for himself or herself.[4] The rationale for this, according to Beauchamp and Childress, is that it would be “unfair to deprive an incompetent patient of decision-making rights merely because he or she is no longer autonomous”.[5] This emphasis on preserving patient self-determination emerged in the years following the Belmont Report, when the report's principle of respect for persons was rearticulated as a mandate to respect a patient's autonomy.[6] Since then, important court cases have advanced substituted judgment as a means for accomplishing this goal. For example, in the Earle N. Spring case, the court applied the substituted judgment principle to an incompetent patient who, when competent, had not clearly expressed wishes for or against treatment, but whose family believed he would have chosen to discontinue treatment.[7]

By promoting patient autonomy even for those who can no longer express their own wishes, the substituted judgment standard departs from earlier ethical norms. For centuries, medical ethics in the West was rooted in the virtue ethics of the Hippocratic corpus, Greek philosophers, and Christian writers. When presented with a medical decision, physicians themselves were expected to discern the right and good thing to do.[8] In the latter part of the twentieth century, U.S. medical ethics shifted its focus to maintaining four principles: nonmaleficence, beneficence, autonomy, and justice.[8] Of these principles, autonomy is the one least emphasized in traditional norms, but it has gradually become the leading principle of clinical ethics.[8], [9] As a result, doctors are now taught that they are not to ask what is best for patients who can no longer express their wishes, but rather are to respect such patients' autonomy even when that autonomy cannot be expressed by the patients themselves.[10] Thus medical ethics has shifted from emphasizing best interest to emphasizing autonomy; from informed consent to patient choice.[9]

When medical students encounter the principle of substituted judgment, and learn that they are not to apply a best interest standard to medical decisions for incapacitated patients, they may not recognize the significance of this historical paradigm shift, nor easily abandon traditional ethical constructs and internalize the new system. If students do not internalize the ethical principles they are being taught, they may not practice according to those principles when they enter the professional world. To examine this subject further, we reviewed the ways twenty-nine medical students analyzed the ethical dimensions of medical decision-making for a patient who had lost decisional capacity. Our goal was to describe ways that students comprehend, and potentially miscomprehend, the substituted judgment standard and the principles behind it.

## Methods

The final assignment in the quarter-long (15 classroom hours) Doctor-Patient Relationship course at the University of Chicago Pritzker School of Medicine asks first year students to analyze the ethical issues raised in one of three cases presented by “Intensive Care,” a NBC Dateline television program aired August 9, 1996.

One case involved a middle-aged, homeless woman who was brought to the emergency room after suffering a massive intracranial hemorrhage. She was profoundly obtunded, possessed medication for schizophrenia, and showed signs of a previous stroke. The clinicians who evaluated her believed that medical interventions could preserve her life, but that it was very unlikely that she would recover much, if any, functional or communicative capacity. Faced with the decision of whether to operate or institute palliative care, the medical team tried in vain to locate family members. Members of the team believed surgical intervention was not in the best interest of the patient, but they decided to perform the procedure anyway because their knowledge of public opinion data (invoked as a proxy surrogate) suggested that most families of patients in similar clinical scenarios would choose medical intervention. After an emergency craniotomy was performed, the patient's son and daughter were located, and both reported that their mother would not have chosen to undergo the operation had she been able to express her wishes. The patient never regained consciousness and died five months later of complications.

Medical students were asked to write a brief essay about this vignette according to the following instructions: “Discuss how surrogate decision-making differs from routine decision-making. Reference the textbook and other class readings. Should physicians give equal weight to an individual's decisions for herself/himself compared to a surrogate's decisions for the individual? Give an example.”

For the purposes of this analysis, students who completed the course in 2005 were emailed and asked for permission to study their essays after removing all identifying information; 104 students were asked, 77 consented to have their essays included, 26 did not respond, and one declined. The study data includes the 29 essays which addressed the vignette described above. The remaining 48 essays were excluded from this study because they discussed a different vignette altogether.

Through an iterative process of textual analysis, the authors reviewed the essays looking for patterns in the way students described, interpreted, and applied the substituted judgment standard. First, two investigators (JF, FC) each read through several essays, coding them for themes and patterns. They then met together to develop a codebook specifying the prominent concepts and themes. JF and RL coded all twenty-nine essays according to the codebook, adding new codes where they emerged. Some passages had multiple implications and were given more than one code. FC and RL then cut and pasted relevant coded parts of the text into an outline of the prominent themes (resulting in over 30 pages of quotations organized by theme). Finally RL synthesized the findings into the concise presentation that follows—emphasizing, and providing quotations to illustrate, the most prominent patterns and themes (individual essays are indicated using the notation E1 to E29).

This process of textual analysis employed three principles that help to strengthen the credibility of the findings and to guard against inaccurate or arbitrary interpretations of textual data. First, following the principle of *constant comparison*, the investigators examined each transcript in relation to the others to ensure that the codebook and our evolving interpretation of the findings reliably followed from the data.[11] Second, different investigators read the data and collaborated to come up with a shared interpretation. Bringing to bear multiple perspectives in data analysis and interpretation strengthens the credibility of the findings and is known as investigator *triangulation*.[12] Third, although we reviewed all 29 essays, we reached *theoretical saturation*—the point at which subsequent essays did not reveal substantial new themes[13]—after coding roughly half of the essays.

## Results

Students accurately reproduced the ethical principles taught in the course. They espoused the primacy of patient autonomy, saying *“patient preferences must always come first”* E29. They correctly summarized substituted judgment, indicating the surrogate *“…must make decisions that best approximate what the patient preferences would have been…”* E2. And many directly cited the class's primary text which warned, *“Surrogates must be careful to avoid the common ethical pitfall of injecting their own values and beliefs into the decision-making process, as only the patient's values and beliefs are relevant to the decision”* (p85).[14] Thus, students appeared to be able to recapitulate the principles and use the standard vocabulary they were taught.

However despite explicitly endorsing substituted judgment, with its accompanying principles of autonomy and patient self-determination, students tended to judge the physicians' actions by standards which are not consonant with the substituted judgment standard. For example, students expected the physicians, in their role as decision makers, to balance the patient's wishes with the desires of the family. One student claimed,

*… the possibility of the patient persisting in a vegetative state could prove burdensome for the surrogates should they assume responsibility for care of the patient upon release from the hospital. [Thus]… doctors should equally consider both the wishes of patients and those closest to them.* E13

Another wrote, *“…one of the physicians' main goals was doing what they thought the family would want; that is pleasing the family”* E3. Students did not seem to recognize that for physicians to make decisions based on the family's wishes or the physicians' own judgments about the family's needs is not compatible with an ethic of substituted judgment and patient self-determination.

Substituted judgment is based on patient autonomy,[5] yet while students agreed that patient autonomy must guide decisions, they often steered rhetorically toward the notion that best interest should be the primary guide for physicians and surrogate decision makers. For example, concerning the decision to invoke public opinion data as a proxy surrogate, one student commented, *“I wonder if using this rationale to make a decision was truly based on what was in the best interest of the patient”* E11. Another stated, *“Ultimately, as many of the physicians in the video stated, the surrogate must decide what is in the patient's BEST INTEREST”* (emphasis in original) E23. Others presented substituted judgment as subordinate to best interest, saying *“If… the physician thinks that the surrogate is not truly considering the patient's best interest, then it might be unethical to fully weigh-in [the surrogate's] decision”* E3.

Along these lines students at times found it perplexing that the substituted judgment standard compelled physicians to make a decision which was not consistent with their judgment of the patient's best interest.

*…the physicians admitted that they made the wrong decision. Rather than respecting the dignity of [the patient], they played it safe and chose to treat when all medical and quality of life issues pointed to withholding life-sustaining treatments. It was very interesting, however, that the physicians admitted that they would probably choose the same action again, despite acknowledging that it was wrong.* E3

Throughout these various appeals to best interest, students did not seem to be intentionally challenging the importance of autonomy. Rather, they seemed to forget their earlier statements about the need to let patient autonomy govern all medical decisions. Ironically, some claimed that autonomy becomes central primarily when a patient cannot express his or her wishes.

*In most cases, one would expect the goal to be improving the quality of life of the patient, generally by improving their health… [but when] a patient presents in a coma necessitating life support to prolong life, with little chance of recovery, the ultimate goal of the medical team becomes respecting the patient's preferences.* E22

Students did not always understand the rationale behind the decision to treat. Many failed to recognize the use of public opinion data as an attempt to preserve patient autonomy. Thus one wrote, *“Certainly in this case, had her preferences been known, the doctors surely would have respected the patient's autonomy. Instead, the physicians made up a family from public opinion data”* E15. Students who misunderstood the rationale explained the decision by suggesting doctors based it on implied consent E9, the inability to locate family members who objected E7, and medical indications E7. Others suggested doctors may have been biased by the patient's history (stroke, schizophrenia), the patient's current status (homeless, uninsured), the fear of lawsuit and the irreversibility of non-treatment, popular opinion favoring treatment, hope of giving the patient a chance at recovery, and the desire to give the family time to come to terms with the situation. Several students endorsed the decision not because it preserved autonomy, but because they believed physicians must always try to preserve life E20, or must always err on the side of life when there is uncertainty E11.

When evaluating the decision to treat the patient, many students did not endorse the idea that the decision was based on a procedural ethic, that is, an approach whose merits are determined by the process utilized rather than the endpoint reached. The language of “right” and “wrong” was frequently applied based on students' judgments of the decision's outcome, and whether the decision matched what were later found to be the patient's expressed preferences. This shows students were focusing on the result of the decision, rather than its method.

## Discussion

After a course on medical ethics, medical students still tended to misunderstand what the substituted judgment standard requires. In some cases their use of the right words masked a deeper misunderstanding of the concepts at play, suggesting the possibility that the substituted judgment vocabulary was acting as something of a rhetorical façade: a publicly appealing front covering a less acceptable construct. For these students, the terminology gave the appearance of appealing to substituted judgment standard even as the substance of the student's valuation was not compatible with that standard.

In reference to a patient who no longer had decisional capacity, students commonly suggested that the patient's doctors should make decisions based on their own judgments of the patient's and/or the family's best interest. As such, they tended to misrepresent the ethical reasoning behind others' decisions and judge those decisions based on outcomes rather than procedures. These findings suggest that although the students could recapitulate the ethical doctrines presented in the course, they did not realign their ethical concepts to be in accordance with them. This raises questions about whether training in ethics actually changes how doctors arrive at decisions, or only changes physicians' vocabularies and the ways they defend their decisions. This also raises questions about whether substituted judgment is a particularly problematic standard for students to internalize.

Admittedly this study is limited by its small sample size, and its focus on students in one class of one medical school. Thus some might dismiss the trend as a quirk of a particular context or might suggest that students' misapprehensions are a developmental phenomenon which will be overcome by the process of medical education. Perhaps by the time these students become practicing physicians they will have internalized the ethics of autonomy and substituted judgment along with the other prominent principles of contemporary medical ethics. Additionally it is possible that students comprehend the ethic, but struggle to write about it with clarity.

Along these lines it is worth noting that qualitative studies among practicing physicians have shown some trends that parallel those we observed among medical students. After interviewing 20 intensive care physicians, Alexia Torke and colleagues described physicians actively balancing patients' wishes against other considerations, such as the physician's view of the patient's best interest, or the surrogate's needs and wishes. Also, physicians in their study often allowed clinical and ethical reasoning to combine and overlap, such that physicians were drawing on both clinical and ethical knowledge without making clear distinctions between the two realms.[15] (This latter trend is reminiscent of students in our study who defended the decision based on medical indications.) If students receive their clinical training from faculty who do not actively and accurately implement substituted judgment, the students are less likely to have misconceptions challenged and corrected.

An alternative consideration is whether there is something inherent to the ethic of substituted judgment that makes it difficult to internalize. For many students and professionals, it may run counter to their moral intuitions, therein making synthesis difficult. Additionally, some scholars have noted problems with the substituted judgment standard: patients often do not have pre-determined preferences,[9] patients' preferences may involve misunderstandings of medical factors,[9] previously articulated wishes may be hard to interpret in a given scenario,[9], [16] physicians can sway the decision making process by the way they present information,[9] and surrogates may have ulterior motives.[17] In addition, a body of empirical studies has shown that surrogates are often poor predictors of patient wishes. [1], [3], [9], [18], [19], [20]

Conceptually the substituted judgment standard is clouded by problematic presuppositions. It is not self-evident to all that substituted judgment is morally comparable to self-determination,[10] that autonomy and self-determination continue to have meaning when severe brain-damage has occurred,[17], [21] or that patients value self-determination as much as contemporary ethicists do.[2], [9], [22] Moreover, some worry that the “absolutization” of autonomy may “override good medical judgment, encourage moral detachment on the part of the physician, and even work against the patient's best interests” (p 1160).[8] To the extent students share these concerns, they may find it difficult to internalize substituted judgment and its related ethical principles.

Students might have an easier time with the principle if more attention were given to its strengths and limitations, and the contexts in which it is appropriate and inappropriate as a guiding principle. For example, Allen Buchanan and Dan Brock recommend surrogate decision makers first look for advance directives (documented or by proxy), but claim “where there is no advance directive, there is no one guidance principle appropriate for all cases” (p 113).[23] They recommend employing the principle of substituted judgment when patient preferences are clear but being guided by a principle of best interest when such preferences are unclear. Presenting a more nuanced view of substituted judgment may help students understand more clearly its underlying assumptions and the situations in which it is most appropriate.

Finally, we cannot overlook the possibility that students' ambiguity toward substituted judgment stems not from misunderstanding, but rather an awareness of the moral complexity of the situation, and an appreciation for the competing moral principles at work. However we consider this less likely since the essays contain little to no direct discussion about the limitations of substituted judgment in general or the appropriateness of its use in this setting. Rather, students tended to give superficial endorsement to the principle and then analyze the situation using a different set of principles.

Substituted judgment remains the standard approach to making decisions for patients who lack decisional capacity and do not have adequate advance directives, because it preserves the contemporary emphasis on patient autonomy as the first principle of medical ethics. However, in light of students' inability to internalize the ethic, it is worth considering whether the principle benefits patients and the practice of medicine to the degree intended. If student physicians view themselves as decision makers committed to serving the best interests of incapacitated patients and their families, then perhaps medical ethics educators should build on that disposition by promoting models for decision-making which take beneficence as their first principle, while clarifying the dangers that follow if patients' expressed wishes are not sufficiently taken into account.
